# 65. In Vivo Efficacy of Human Simulated Minocycline (MIN) against *Stenotrophomonas maltophilia* (STM)

**DOI:** 10.1093/ofid/ofab466.065

**Published:** 2021-12-04

**Authors:** Andrew J Fratoni, David P Nicolau, Joseph L Kuti

**Affiliations:** 1 Hartford Hospital, Hartford, Connecticut; 2 Center for Anti-Infective Research and Development, Hartford Hospital, Hartford, Connecticut

## Abstract

**Background:**

The current susceptibility breakpoint for MIN against STM is 4mg/L, yielding >99% of isolates susceptible. Unfortunately, there are limited pre-clinical and clinical data to support this breakpoint for STM. The purpose of this study was to evaluate the efficacy of a MIN human simulated regimen (HSR) against STM across a wide range of MICs in the murine neutropenic thigh model.

**Methods:**

Clinical STM with modal MIN MICS of 0.25-8mg/L were included. Confirmatory pharmacokinetic (PK) studies were performed in infected neutropenic mice to develop a MIN HSR providing an area under the curve (AUC) and maximum concentration (Cmax) exposure similar to MIN 100mg intravenous (IV) q12h at steady-state based on PK parameters from critically ill adult patients. The murine neutropenic thigh infection model was utilized to examine the antibacterial effects of the confirmed MIN HSR against 17 STM. Both thighs of neutropenic ICR mice were inoculated with bacterial suspensions of 10^7^ colony forming units (CFU)/mL. Two hours after inoculation, the MIN HSR was administered subcutaneously (SC) over 24h. Control mice received normal saline. Efficacy was measured as the change in log_10_CFU/thigh at 24h compared with 0h controls.

**Results:**

MIN 22, 10, 14, and 10mg/kg dosed SC at 0, 6, 12, and 18h best recapitulated the human Cmax and AUC profile. Mean ± standard deviation bacterial burden at 0h across all isolates was 6.03±0.32 log_10_CFU/thigh. Bacterial growth was 1.35±0.68 log_10_CFU/thigh in 24h controls. Six of 7 isolates (86%) with MIC ≤ 0.5mg/L achieved 1-log kill with MIN HSR (-1.44±1.37 log_10_CFU/thigh). All STM with MIC ≥ 1mg/L experienced bacterial growth (1.18±0.79 log_10_CFU/thigh) (Figure).

Figure. Efficacy of a minocycline human simulated exposure of 100mg intravenous Q12h in the murine neutropenic thigh model against 17 clinical Stenotrophomonas maltophilia isolates

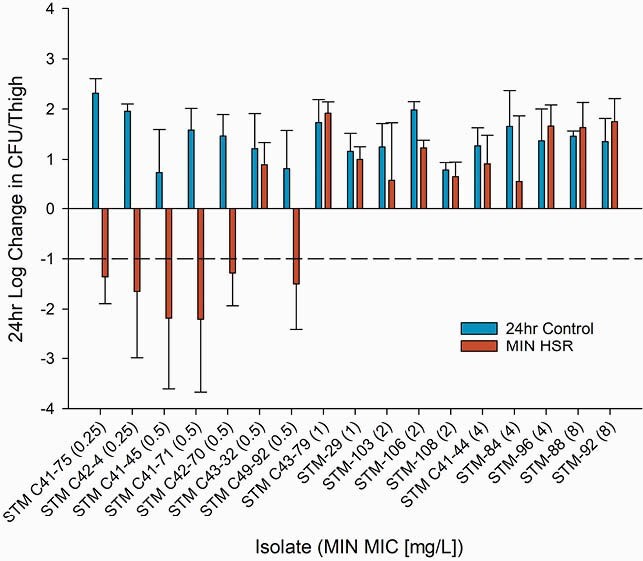

**Conclusion:**

These data describe the in vivo efficacy of a MIN HSR with exposures similar to MIN 100mg IV q12h in critically ill adults. Lack of antibacterial activity against STM with MICs ≥ 1mg/L justifies a reassessment of the current susceptibility breakpoint.

The study was funded under FDA Contract 75F40120C00171.

**Disclosures:**

**David P. Nicolau, PharmD**, **Abbvie, Cepheid, Merck, Paratek, Pfizer, Wockhardt, Shionogi, Tetraphase** (Other Financial or Material Support, I have been a consultant, speakers bureau member, or have received research funding from the above listed companies.) **Joseph L. Kuti, PharmD**, **Allergan** (Speaker’s Bureau)**BioMérieux** (Consultant, Research Grant or Support, Speaker’s Bureau)**Contrafect** (Scientific Research Study Investigator)**GSK** (Consultant)**Merck** (Research Grant or Support)**Paratek** (Speaker’s Bureau)**Roche Diagnostics** (Research Grant or Support)**Shionogi** (Research Grant or Support)**Summit** (Scientific Research Study Investigator)

